# *Primula xideensis* (Primulaceae), a New Species from Sichuan, China, Based on Morphological and Molecular Evidence

**DOI:** 10.3390/plants15121829

**Published:** 2026-06-12

**Authors:** Jiang-Tao Li, Xiong Li, Cheng-Wu Liu, Bo Xu, Jun Hu, Fan-Juan Meng, Wen-Bin Ju

**Affiliations:** 1 Jilin Provincial Key Laboratory of Tree and Grass Genetics and Breeding, College of Forestry and Grassland Science, Jilin Agricultural University, Changchun 130118, China; 15378658484@163.com; 2 Mountain Ecological Restoration and Biodiversity Conservation Key Laboratory of Sichuan Province, Chengdu Institute of Biology, Chinese Academy of Sciences, Chengdu 610213, China; lixiong20@mails.ucas.ac.cn (X.L.); liuchengwu23@mails.ucas.ac.cn (C.-W.L.); xubo@cib.ac.cn (B.X.); hujun@cib.ac.cn (J.H.)

**Keywords:** *Primula* sect. *Aleuritia*, morphological taxonomy, chloroplast genomics, molecular phylogeny

## Abstract

We document and illustrate *Primula xideensis*, a new species collected from Xide County, Sichuan Province, China. Morphologically, it resembles *P. stenocalyx*, *P. farinosa s.l.*, and *P. pulchella*, but differs by its roots densely covered with multicellular hairs; elliptic to obovate leaves with non-revolute, irregularly and deeply dentate margins, both surfaces efarinose, shortly hairy, and scabrous; tubular calyx parted to the middle; corolla mouth densely white-farinose; stamens positioned at mid-corolla tube in pin flowers and near the throat in thrum flowers; styles reciprocally placed; and an oblong capsule shorter than the persistent calyx. To clarify its phylogenetic placement, we constructed phylogenetic trees using two datasets: 63 complete chloroplast genome sequences and 75 nuclear ribosomal DNA internal transcribed spacer (nrITS) sequences. Both trees showed similar topologies, consistently placing the new species within a monophyletic group of Sect. *Aleuritia*, supporting its assignment to this section. For a deeper comparison between the new species and other members of Sect. *Aleuritia*, we incorporated chloroplast genomes of seven additional species from this section. The results revealed highly conserved chloroplast genomes among all eight species, with only minor differences between the new species and the others. *Primula xideensis* is currently known only from its type locality. Based on IUCN Red List criteria, its conservation status is assessed as Data Deficient (DD).

## 1. Introduction

Species, as the fundamental units of biodiversity, form the basis of taxonomy and conservation biology [1]. However, the species concept itself remains fluid, with numerous lineages undergoing rapid diversification. Traditional morphological approaches and modern molecular systematics each have limitations in species delimitation: the former is susceptible to convergent evolution, while the latter may lead to excessive splitting [2,3,4]. Consequently, integrative taxonomy, which synthesizes multiple lines of evidence, has become the prevailing paradigm. Within this methodological framework, the genus *Primula* (Primulaceae) serves as a classic model for studying evolutionary processes such as heterostyly and adaptive radiation, making its taxonomy exemplary [5]. The genus is widely distributed across temperate and alpine regions of the Northern Hemisphere, with the Hengduan Mountains in southwestern China representing its global center of modern diversity and speciation, hosting approximately 500 species with remarkably high endemism [6,7,8,9]. Although research on *Primula* has progressed from traditional morphology to an integrative phase, core taxonomic challenges persist. In diversity hotspots like the Hengduan Mountains, habitat fragmentation and recent rapid diversification have given rise to numerous morphologically continuous species complexes, likely harboring substantial cryptic diversity [10,11]. Furthermore, phylogenetic relationships based on limited gene loci remain conflicting or poorly resolved in some groups, and the infrageneric classification (e.g., sect. *Aleuritia*) requires further clarification [12]. Therefore, integrating morphological and molecular phylogenetic evidence to conduct fine-scale studies on specific lineages in these critical regions is essential for discovering new species and elucidating their evolutionary history.

Sect. *Aleuritia* was formally established by Duby (1844) and subsequently refined through taxonomic revisions [13]. This section primarily comprises a series of perennial species, typically farinose or efarinose, with a leaf rosette base lacking scales. Leaf morphology varies from entire to dentate or lobed. The key diagnostic features are concentrated in the inflorescence structure: an umbel (rarely a solitary flower) borne on a scaleless scape, with bracts often swollen at the base into a sac-like form or decurrent into auriculate projections. The flowers are predominantly heterostylous, with a funnel-shaped corolla bearing a ring-shaped appendage at the throat, a campanulate to tubular calyx, and a coriaceous, tubular or subglobose capsule [14]. The type species of the section—*Primula farinosa* L.—defines its core morphological framework. Currently, species within this section are mainly distributed across temperate and alpine regions of the Northern Hemisphere [6]. They exhibit particularly high species diversity in Asian high-mountain areas, especially from the Himalayas to the Hengduan Mountains, and typically inhabit meadows, scree slopes, or alpine scrub communities.

**Figure 1:**
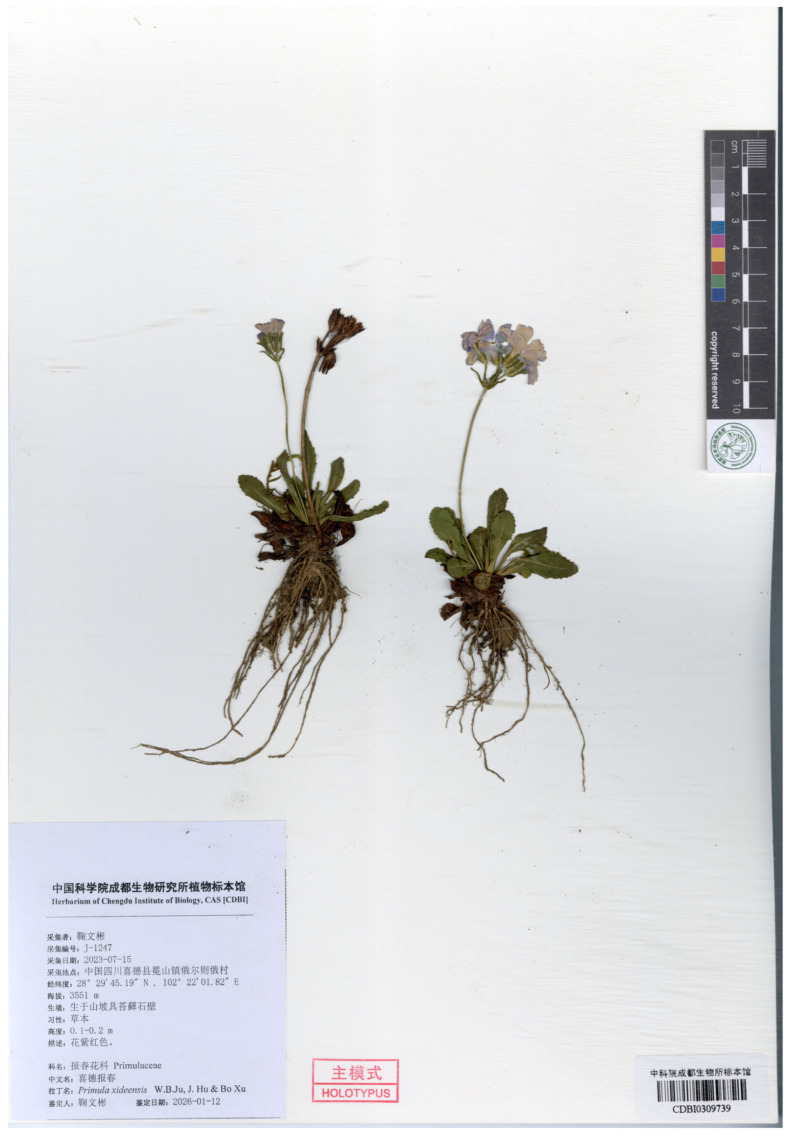
Specimens of *Primula xideensis* (The herbarium label includes Chinese terms with their English translations: “中国科学院成都生物研究所植物标本馆” = Herbarium of Chengdu Institute of Biology, CAS; “采集者” = Collector; “鞠文彬” = W.B. Ju; “采集编号” = Collection No.; “采集日期” = Date; “采集地点” = Locality; “中国四川喜德县冕山镇俄尔则俄村” = Erze’e Village, Mianshan Town, Xide County, Sichuan, China; “经纬度” = Coordinates; “海拔” = Elevation; “生境” = Habitat; “生于山坡具苔藓石壁” = on mossy rock slope; “习性” = Habit; “草本” = herb; “高度” = Height; “描述” = Description; “花紫红色” = flowers purplish-red; “科名” = Family; “报春花科” = Primulaceae; “中文名” = Chinese name; “喜德报春” = *Primula xideensis* W.B.Ju, Hu Ju, Bo Xu *sp. nov*; “拉丁名” = Scientific name; “鉴定人” = Identified by; “鉴定日期” = Date of identification; “主模式” = Holotype; “中科院成都生物所标本馆” = Herbarium of CDBI; “CDBI0309739” = accession number. The Latin term “HOLOTYPUS” indicates that this specimen is the holotype.).

**Figure 2:**
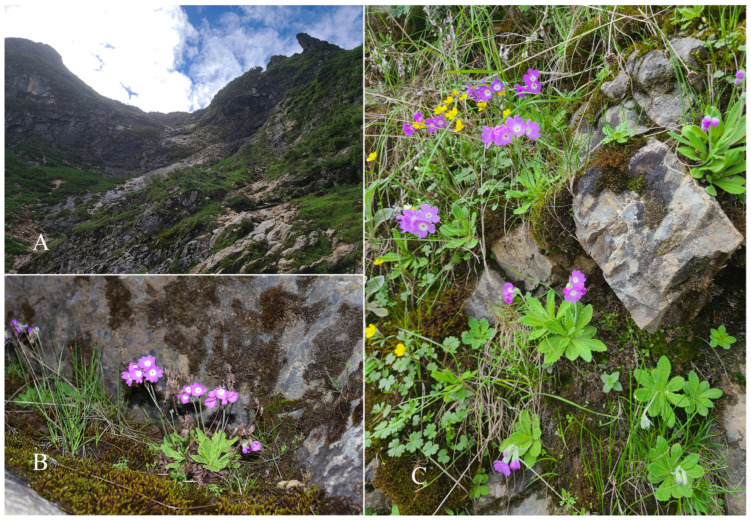
Habitat of the *Primula xideensis* sp. nov. (**A**–**C**).

**Figure 3:**
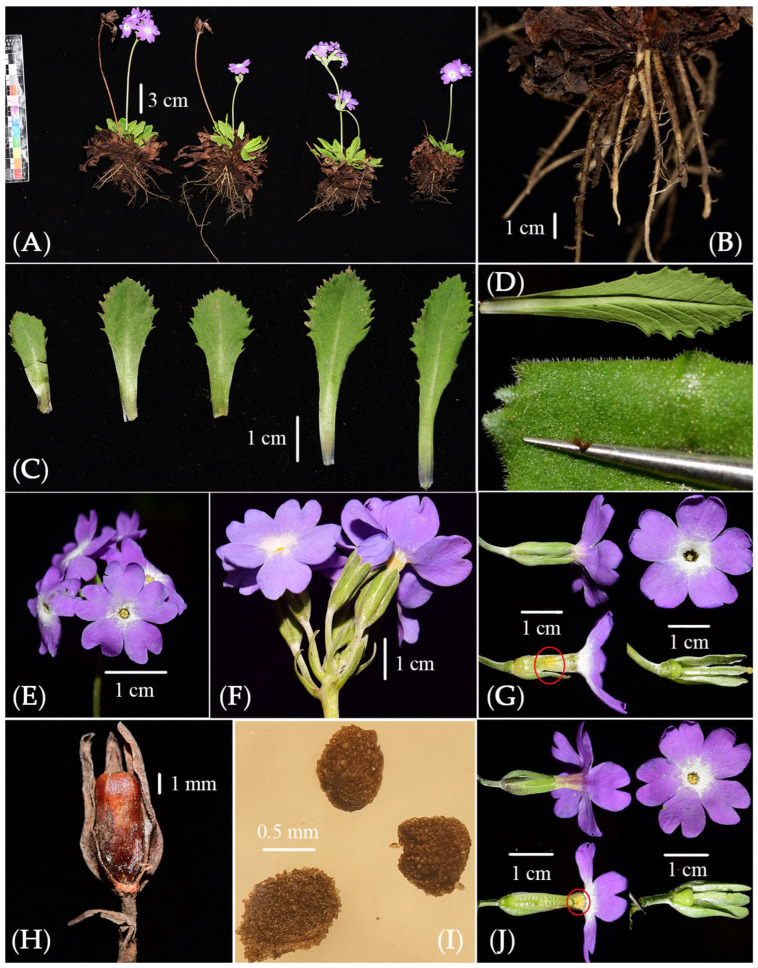
*Primula xideensis* sp. nov. (**A**) fresh plants; (**B**) plant base, showing the roots and withered old leaves. (**C**) leaves; (**D**) Leaf abaxial surface and indumentum; (**E**) Corolla; (**F**) Bracts and calyx; (**G**) pin flower; (**H**) capsule; (**I**) seed; (**J**) thrum flower.

**Figure 4:**
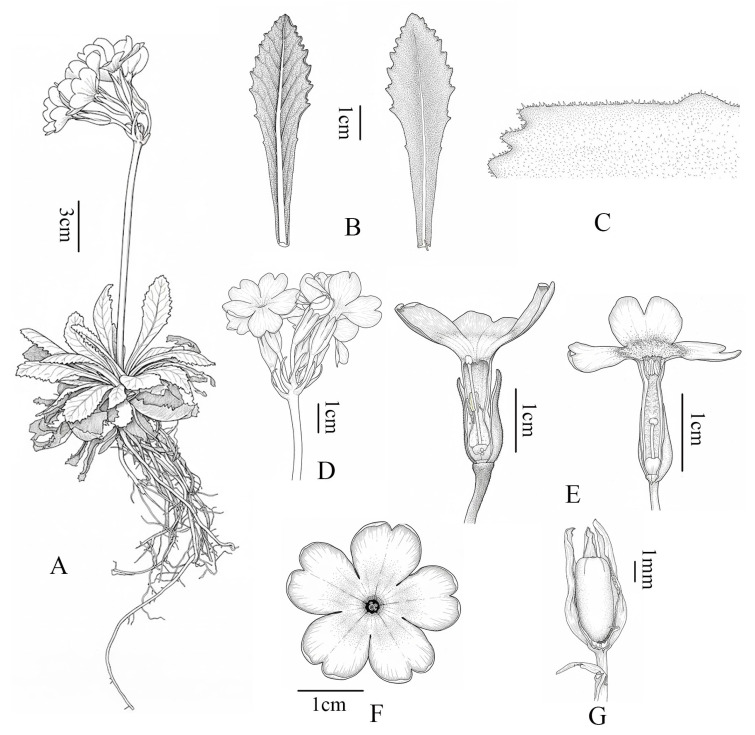
*Primula xideensis* sp. nov. (**A**) habit; (**B**) leaves; (**C**) Leaf indumentum; (**D**) Bracts and calyx; (**E**) flowers: pin flower, thrum flower; (**F**) front of the flower; (**G**) capsule.

In July 2023, during a floristic survey in southwestern China, we encountered a distinctive population of *Primula* exhibiting unique morphological traits at Ejize’e Village, Mianshan Town, Xide County, Sichuan Province (coordinates: 28°29′45.19″ N, 102°22′01.82″ E). The plants bear an umbellate inflorescence with numerous flowers, bracts typically swollen at the base, and heterostylous flowers, which are characteristic of sect. *Aleuritia*. However, this taxon is markedly distinguished from other members of the section by a combination of key features: its leaves are densely covered with coarse hairs (scabrous), the corolla mouth is densely farinose (coated with white farina), and the pedicel equals the corolla tube in length (Figure 1, Figure 2, Figure 3 and Figure 4). To thoroughly document this plant, we conducted follow-up investigations in the type locality and adjacent areas from July 2023 to November 2024, collecting voucher specimens for detailed examination. Through comprehensive morphological analysis, comparison with relevant literature and herbarium specimens of related taxa, and the construction of molecular phylogenetic trees based on nuclear ribosomal DNA internal transcribed spacer (nrITS) and chloroplast genome data, we initially concluded that this entity represents a previously undescribed species. To further verify the distinctiveness of this new species from a chloroplast genomic perspective, we downloaded the complete chloroplast genome sequences of seven other species of sect. *Aleuritia* from the NCBI GenBank database and performed comparative genomic analyses with the newly sequenced chloroplast genome of the candidate new species. These analyses included comparisons of genome structure, gene order, GC content, and sequence divergence patterns. The results showed that the chloroplast genomes of the eight species are highly conserved, but the new species exhibited stable and distinguishable nucleotide differences in nucleotide diversity (Pi) and relative synonymous codon usage (RSCU) value. These chloroplast genomic differences, together with morphological characters and nrITS phylogenetic evidence, strongly support the recognition of this taxon as a new species, which we hereby formally describe with a detailed morphological description, molecular evidence, and illustrations.

## 2. Results

### 2.1. Morphological Observation and Comparison

Observation of both fresh and dried field specimens indicates that the new species is morphologically similar to *P. stenocalyx*, *P. farinosa s.l.* and *Primula pulchella Franch.* These taxa share a similar general habit, rhizome thickness, plant size, inflorescence structure, flower number, and heterostyly. The new species resembles *P. stenocalyx* in having narrowly lanceolate bracts and a tubular calyx, but differs in its roots densely covered with multicellular hairs, leaves elliptic to obovate, both surfaces efarinose, densely covered with short hairs and scabrous, margins dentate to irregularly deeply dentate and not revolute, the mouth of the corolla tube densely white-farinose, pin flowers with stamens inserted about the middle of the corolla tube and styles reaching the corolla throat, thrum flowers with stamens positioned near the corolla throat and styles inserted about the middle of the corolla tube, and an oblong capsule shorter than the persistent calyx. Although both the new species and *P. farinosa s.l.* are heterostylous and may have efarinose leaves, the former differs in having roots densely covered with multicellular hairs, leaves elliptic to obovate with dentate to irregularly deeply dentate, non-revolute margins, leaf surfaces densely short-hairy and scabrous, bracts narrowly lanceolate and as long as or slightly shorter than the pedicels, a tubular calyx, a corolla tube 8–16 mm long with the mouth densely white-farinose, and stamens and styles differing in position from those of *P. farinosa s.l.* The new species also differs from *P. pulchella*, although both possess heterostylous flowers and oblong capsules, in its roots densely covered with multicellular hairs, leaves elliptic to obovate, efarinose, densely short-hairy and scabrous, margins dentate to irregularly deeply dentate and not revolute, bracts narrowly lanceolate and as long as or slightly shorter than the pedicels, a tubular calyx, a corolla mouth densely covered with white farina, and floral organ positions different from those of *P. pulchella*. A detailed morphological comparison is provided in Table 1.

### 2.2. Characteristics of the P. xideensis Chloroplast Genome

**Figure 5:**
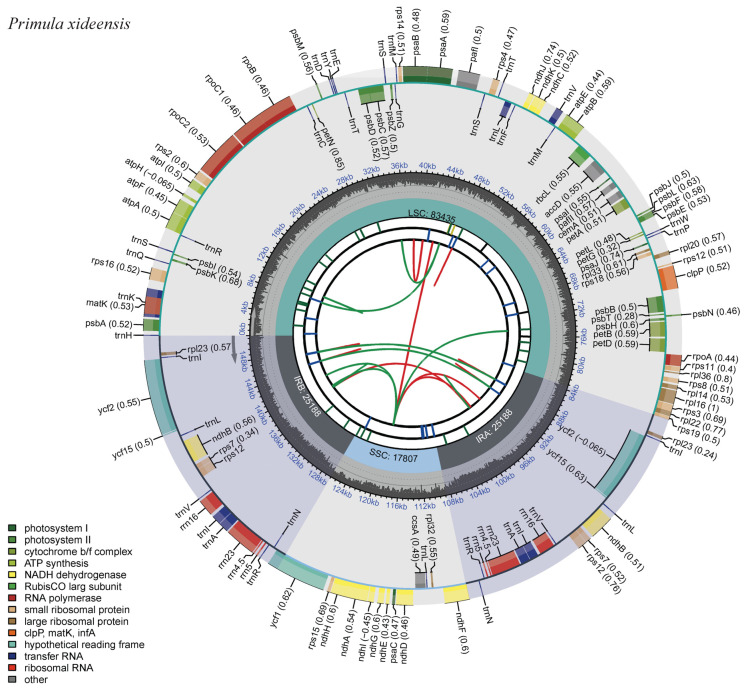
The chloroplast genome map of *Primula xideensis*. The outer circle shows the distribution of genes (different colors represent different roles). The arrows indicate the transcription directions of the genes inside and outside of the circle. The gray inner circle represents the GC content.

**Figure 6:**
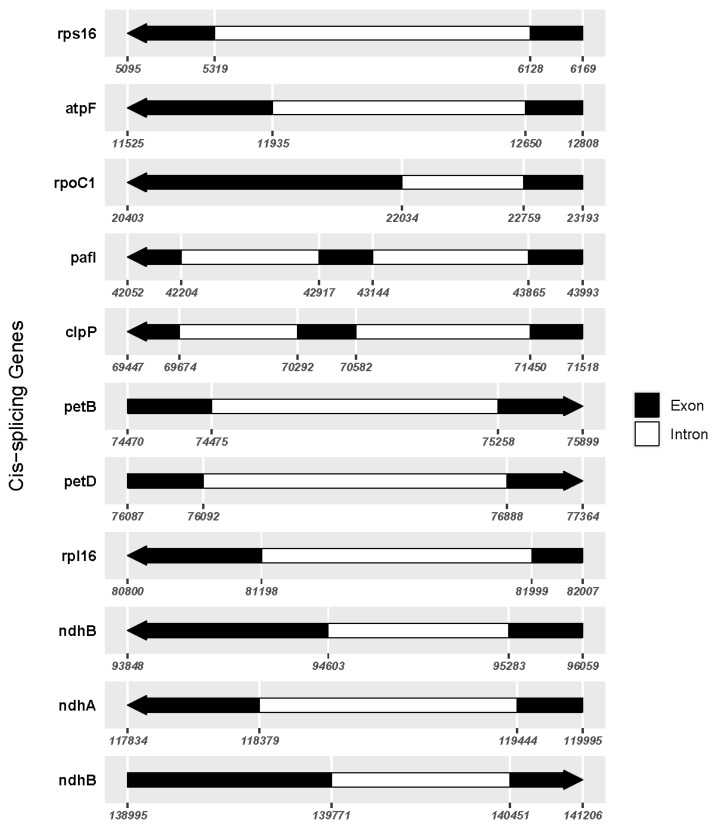
Schematic diagram of a cis-spliced gene in the chloroplast genome, with exons shown in black and introns in white. Arrows indicate the orientation of the gene reading frame.

**Figure 7:**
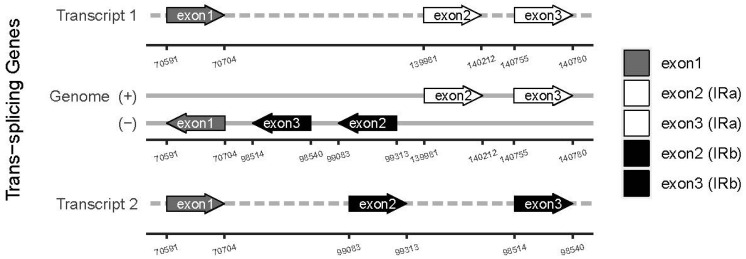
Schematic map of the trans-splicing gene *rps12* in the chloroplast genome. It has three unique exons. Two of them are duplicated as they are located in the IR regions.

The results revealed that these cp genomes exhibit a typical quadripartite structure, comprising a large single-copy (LSC) region, a small single-copy (SSC) region, and two inverted repeat (IR) regions. The eight cp genomes ranged from 153,678 bp (*P. stenocalyx*) to 154,705 bp (*P. pumilio*) in total length, demonstrating a high degree of size conservation (Table 2). The LSC region varied from 83,953 bp to 85,197 bp, the SSC from 17,661 bp to 17,865 bp, and both IR regions were consistently approximately 25,800 bp across all species. The overall GC content was stable at 36.8–37.0%. Notably, the IR regions possessed the highest GC content (approx. 42.7%), followed by the LSC (approx. 34.8%) and then the SSC (approx. 30.2–30.3%). Gene content and order were highly conserved. The chloroplast genome of *Primula xideensis* contains 131 genes, including 37 tRNA genes, 8 rRNA genes, and 86 protein-coding genes (Figure 5; Table 3). Additionally, 15 genes contain one intron, and three genes possess two introns (Figure 6 and Figure 7). The total number of genes ranged from 129 (*P. gemmifera*) to 132 (*P. farinosa s.l.* and *P. stenocalyx*). This included 85–87 protein-coding genes (CDS), 36–37 transfer RNA (tRNA) genes, and consistently 8 ribosomal RNA (rRNA) genes in all eight species. The minor variations in gene number are mainly attributable to the differential presence or annotation of specific tRNAs or small CDSs, which is common within plant genera.

In summary, the chloroplast genomes of these eight *Primula* species are highly conserved in terms of genome size, structural organization, GC content, and gene order. These newly sequenced genomes provide valuable genomic resources for future studies on phylogenetic reconstruction, species identification, and evolutionary biology within the genus *Primula* [15].

### 2.3. Comparative Chloroplast Genome Analysis

#### 2.3.1. Ir Expansion and Contraction

**Figure 8:**
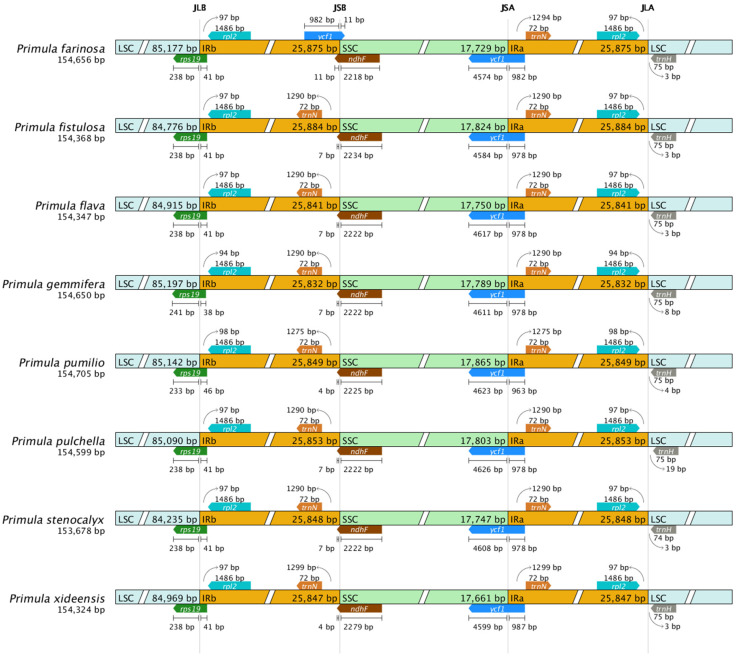
Comparison of LSC, IR, and SSC junction positions among 8 *Primula* species in chloroplast genomes. JLB denotes the LSC/IRb junction, JSB denotes the SSC/IRb junction, JSA denotes the SSC/IRa junction, and JLA denotes the LSC/IRa junction.

Highly conserved inverted repeat (IR) regions play a crucial role in stabilizing the chloroplast genome structure [16]. Comparative analysis of the four IR/SC boundaries (JLB, JSB, JSA, JLA) across the eight *Primula* genomes revealed a generally conserved quadripartite structure, with species-specific variations mainly confined to the extension lengths of *ndhF* and *ycf1* into the IR regions, as well as minor distance differences at JLA (Figure 8). Notable variations (where expansion/contraction occurred): JSB boundary (IRb/SSC). In all species, JSB lies between trnN (IRb side) and ndhF (SSC side). However, the extent of ndhF protruding into IRb differs markedly: *P. farinosa s.l.* shows the largest expansion (11 bp), six species (*P. fistulosa*, *P. flava*, *P. gemmifera*, *P. pulchella*, *P. stenocalyx*) show a moderate expansion (7 bp), while *P. pumilio* and the new species *P. xideensis* exhibit the smallest expansion (only 4 bp). Thus, relative to the majority, *P. pumilio* and *P. xideensis* have a contracted (shorter) ndhF extension into IRb. JSA boundary (SSC/IRa). The ycf1 gene spans this boundary and extends into IRa. The extension length varies from 963 bp (*P. pumilio*) to 987 bp (*P. xideensis*). Most species show 978 bp (*P. fistulosa*, *P. flava*, *P. gemmifera*, *P. pulchella*, *P. stenocalyx*), with *P. farinosa s.l.* at 982 bp. Consequently, *P. xideensis* possesses the longest *ycf1* extension into IRa (987 bp), representing a notable expansion relative to all other examined species. JLA boundary (IRa/LSC). The distance from the *trnH* gene to the boundary is 3 bp in most species (including *P. xideensis*), but expands to 8 bp in *P. gemmifera* and 19 bp in *P. pulchella*, indicating a local expansion of the intergenic region in these two species.

#### 2.3.2. Synteny Analysis

**Figure 9:**
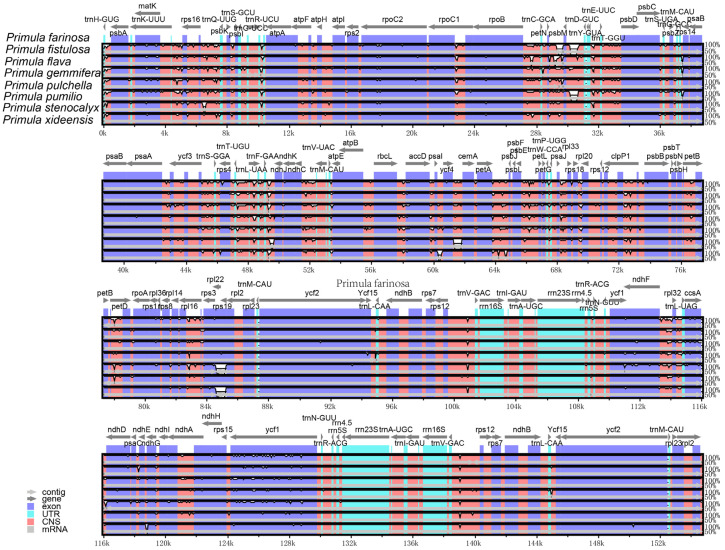
Visualization of genome alignment of the chloroplast genomes of 8 *Primula* species by mVISTA. The *x*-axis represents the coordinate in the chloroplast genome. The *Y*-axis represents different species, and sequence similarity of aligned regions is displayed as horizontal bars, expressed as a percentage within 50–100%.

**Figure 10:**
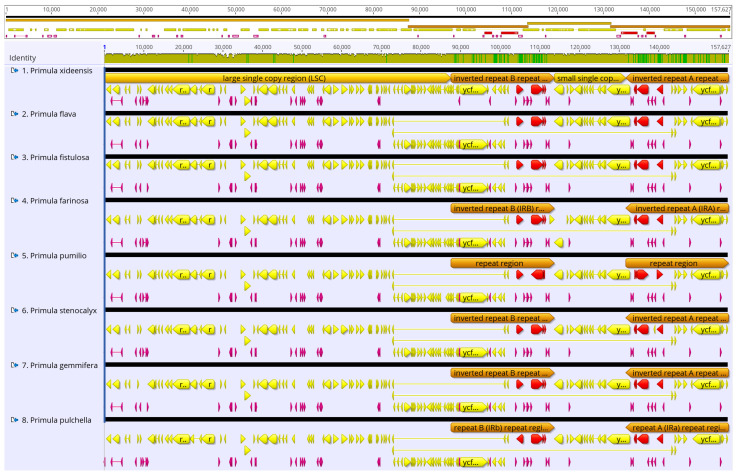
Comparison of the genome structure of 8 *Primula* species using Geneious Prime. Annotations of CDSs, tRNA genes, and rRNA genes are shown in yellow, in red, and with purple-red arrowheads, respectively.

Whole-genome synteny analysis using mVISTA and Geneious revealed that all eight *Primula* chloroplast genomes, including the newly described *P. xideensis*, are highly collinear with the reference *P. farinosa s.l.* genome, displaying no evidence of large-scale rearrangements, inversions, or translocations (Figure 9 and Figure 10). Most coding regions showed 100% sequence identity across species, with only minor local differences observed in a few taxa (e.g., a small insertion in *P. fistulosa*, and marginal similarity variations in *P. flava* and *P. gemmifera*), which do not disrupt the overall syntenic structure. Importantly, the previously identified contraction of *ndhF* expansion (4 bp into IRb) and the maximal expansion of *ycf1* (987 bp into IRa) in *P. xideensis* did not alter gene order or collinearity; rather, they represent length polymorphisms at the IR/SC boundaries that are fully compatible with the conserved synteny observed across the genus. Thus, while *P. xideensis* shares the same structural framework as its congeners, its unique combination of boundary-specific length variations distinguishes it at the sequence level without affecting genome-wide synteny.

#### 2.3.3. Candidate Molecular Markers Identification

**Figure 11:**
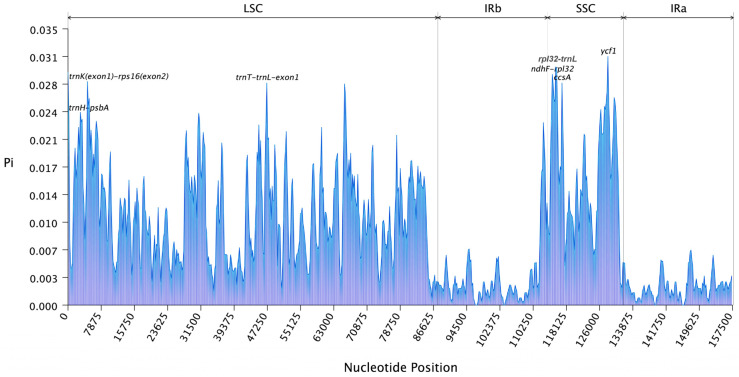
Nucleotide polymorphisms in the chloroplast genomes of 8 species of *Primula* based on sliding window analysis. The window length is 600 bp, and the step size is 200 bp. The horizontal axis indicates the position of the midpoint of a window. The vertical axis indicates the nucleotide polymorphism (Pi value).

Through sliding window analysis, seven hypervariable regions were identified as potential specific molecular markers for species delimitation within *Primula* sect. *Aleuritia* (Figure 11). These regions were mainly distributed in the LSC and SSC regions, with three located in the LSC and four in the SSC. Among the seven hypervariable regions, five were intergenic spacers and two were genic regions. Of these, the *trnK-rps16* intergenic spacer was the longest and contained the most parsimony-informative sites, exhibiting the highest nucleotide diversity (Pi = 0.03137; Table S1). In addition, the *ycf1* gene showed sufficient variability to serve as a molecular marker for species identification and genetic studies in *Primula* sect. *Aleuritia*.

#### 2.3.4. Rscu Analysis

**Figure 12:**
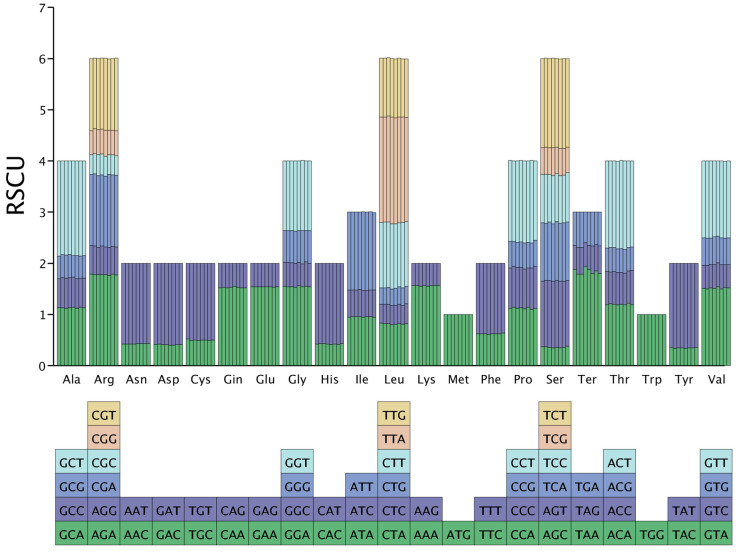
Relative synonymous codon usage (RSCU) values for 8 *Primula* species.

Codons that encode the same amino acid are termed synonymous codons [17]. The relative synonymous codon usage (RSCU) value reflects the ratio between the observed frequency of a codon and its expected frequency under no codon usage bias [18]. The eight *Primula* species showed highly similar codon usage preferences. Analysis revealed that the protein-coding genes of the eight species are encoded by 64 codons (including the three stop codons: UAA, UGA, and UAG; Figure 12). Leucine was the most frequent amino acid. Leucine is encoded by CTG, CTC, CTT, CTA, TTA, and TTG. Among these 64 codons, half end with A/T and the other half with G/C. RSCU (Relative Synonymous Codon Usage) analysis showed that, except for methionine and tryptophan (Met and Trp; RSCU = 1), almost all amino acids are encoded by one to six synonymous codons. Furthermore, 30 codons had RSCU values greater than 1, and 32 codons had values less than 1. In addition, among the 64 codons, exactly 16 end with A, 16 with G, 16 with C, and 16 with T (Table S2). This result indicates that the chloroplast genomes of these eight *Primula* species are relatively conserved and exhibit no significant codon usage bias.

### 2.4. Phylogenetic Relationship

#### 2.4.1. Nrits Data

**Figure 13:**
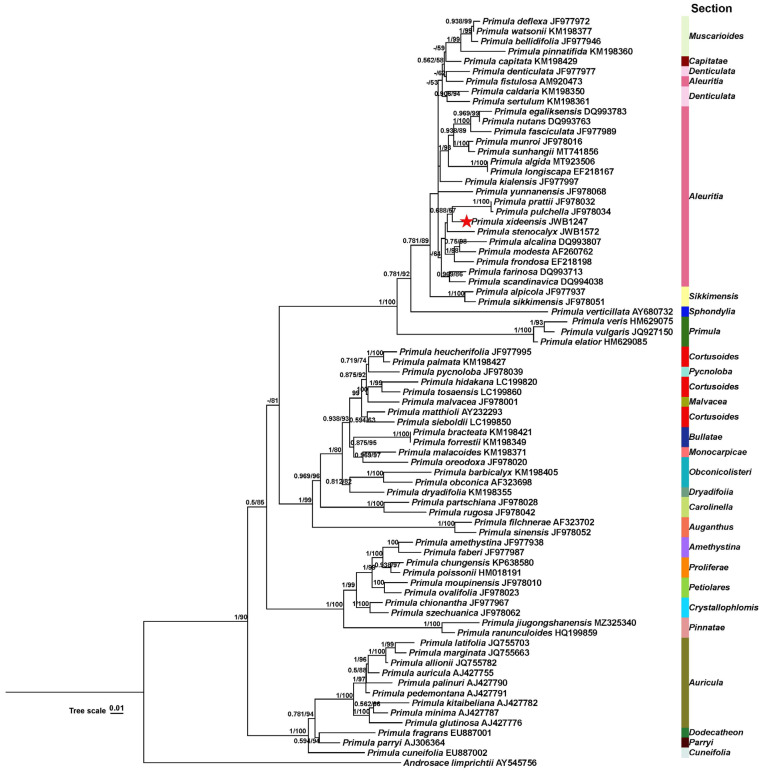
Phylogenetic relationships within *Primula* and its related species were reconstructed using maximum likelihood (ML) and Bayesian inference (BI) based on nrITS. Numbers above branches indicate bootstrap support (BS) from ML analysis and posterior probability (PP) from BI analysis. The phylogenetic position of *Primula xideensis* is highlighted with a red star.

Both the maximum likelihood and Bayesian inference phylogenetic trees constructed based on nrITS exhibited identical topologies with strong nodal support (Figure 13). The new species formed a monophyletic clade with *P. stenocalyx*, *P. pulchella*, and *P. prattii*, which was further nested within section *Aleuritia*. This phylogenetic placement is consistent with the morphological characterization of this section. The discovery of this new species not only enriches the biodiversity of *Primula* in the region but also provides important clues for understanding the evolutionary dynamics and speciation patterns within section *Aleuritia*.

#### 2.4.2. Plastid Data

**Figure 14:**
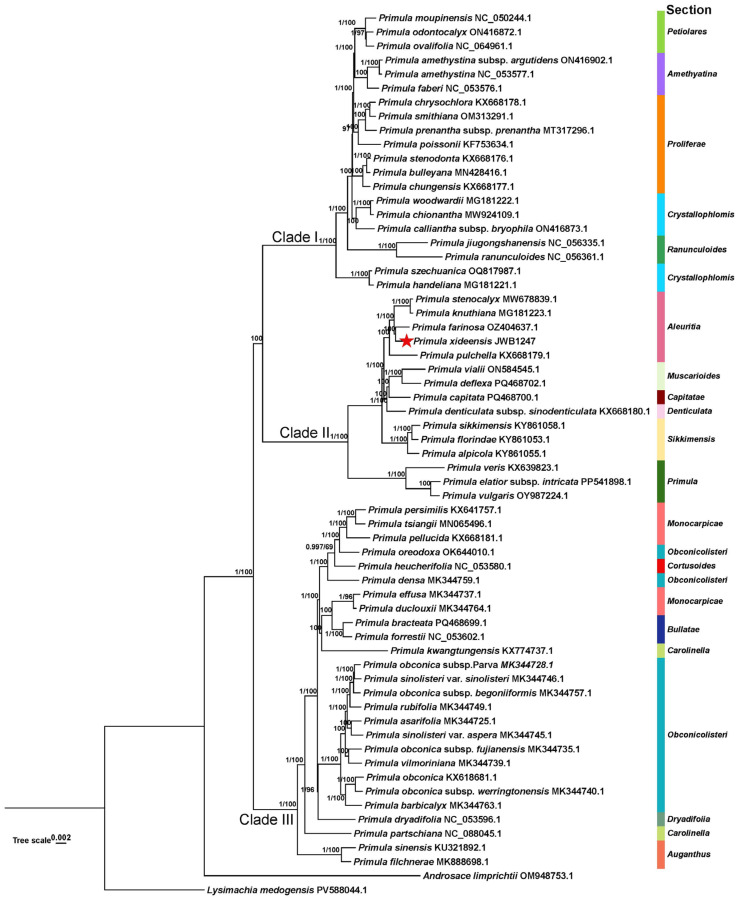
Phylogenetic relationships within *Primula* and its related species were reconstructed using maximum likelihood (ML) and Bayesian inference (BI) based on complete chloroplast genome sequences. Numbers above branches indicate bootstrap support (BS) from ML analysis and posterior probability (PP) from BI analysis. The phylogenetic position of *Primula xideensis* is highlighted with a red star.

The maximum likelihood (ML) and Bayesian inference (BI) phylogenetic trees reconstructed from complete chloroplast genomes exhibited congruent topologies with strong statistical support (Figure 14). Based on the chloroplast phylogeny, the 60 sampled species were divided into three major clades. The newly identified species formed a strongly supported monophyletic group (BS = 100, PP = 91) with *P. farinosa s.l.*, *P. knuthiana*, and *P. stenocalyx*. This clade was further nested within section *Aleuritia*. The phylogenetic placement of the new species based on the chloroplast genome is consistent with that inferred from the nrITS tree, providing further evidence for its classification within section *Aleuritia*.

## 3. Discussion

Phylogenetic trees constructed from the nuclear ribosomal DNA internal transcribed spacer (nrITS) and complete chloroplast genomes both place *Primula xideensis* within Sect. *Aleuritia* with strong support (Figure 13 and Figure 14): in the nrITS tree, the new species forms a monophyletic clade with *P. stenocalyx*, *P. pulchella* and *P. prattii*, while in the chloroplast genome tree, it clusters with *P. farinosa s.l.*, *P. knuthiana* and *P. stenocalyx* (BS = 100, PP = 91). Although the two datasets show minor differences in the exact composition of close relatives, this pattern is consistent with previous findings in the genus. Topological incongruences between nrITS and chloroplast phylogenies in *Primula* have been attributed to differences in evolutionary rates between the plastid and nuclear genomes, as well as frequent introgressive hybridization and incomplete lineage sorting within the genus [19,20]. A recent study on the Hengduan Mountains endemic *P. bella* similarly observed conflicts between chloroplast and nuclear phylogenetic signals, which the authors suggested may result from hybridization events or cytonuclear discordance [21]. These findings indicate that phylogenetic incongruence across different genomic compartments is not uncommon in *Primula*, a rapidly radiating lineage, and should be interpreted as complementary perspectives on the evolutionary history of species rather than as contradictions. Future studies incorporating additional molecular markers, such as single-copy nuclear genes, along with expanded geographic sampling, would help clarify the biogeographic origins and ecological adaptation mechanisms across *Primula* species.

Morphologically, *P. xideensis* exhibits all the core diagnostic traits of Sect. *Aleuritia*, including an umbellate inflorescence, bracts swollen at the base, and heterostyly, which is fully consistent with its phylogenetic placement.

Regarding the related taxon *Primula farinosa*, it should be noted that the typical European *P. farinosa* L. was originally described with crenulate leaf margins. In contrast, the plants previously referred to as *P. farinosa* in the Flora of China (and in this study) have remotely denticulate to nearly entire leaf margins. Following the integrative taxonomic approach, we adopt here the broad sense (*P. farinosa s.l.*) to accommodate this morphological variation.

Notably, the cytonuclear discordance observed in this study actually enriches our understanding of the evolutionary history of the new species: the nuclear signal links it to species such as *P. stenocalyx*, whereas the plastid signal emphasizes its affinity with *P. farinosa s.l.* and other species. This pattern may be linked to frequent hybridization and reticulate evolution within Sect. *Aleuritia* of *Primula* [22].

Furthermore, comparative chloroplast genomics provides additional independent evidence for distinguishing the new species. The chloroplast genome of *P. xideensis* is highly conserved in terms of quadripartite architecture, gene order, and GC content compared to the other seven species in the section, a pattern widely reported in other studies of *Primula* that reflects the highly conserved nature of chloroplast genomes within the genus [23,24]. Nevertheless, stable interspecific differences are observed at the IR boundaries. Notably, *ndhF* shows the shortest expansion into the IRb region (only 4 bp), whereas *ycf1* exhibits the longest expansion into the IRa region (987 bp); these boundary length polymorphisms can serve as molecular characters to distinguish the new species. In addition, sliding window analysis identified seven hypervariable regions (e.g., the *trnK-rps16* intergenic spacer and the *ycf1* gene), which represent promising candidate molecular markers for future species delimitation and population genetic studies within Sect. *Aleuritia*.

Therefore, integrating morphological evidence, nrITS and chloroplast genome phylogenetics, and comparative chloroplast genomics, *P. xideensis* is reliably recognized as a new member of this section.

## 4. Materials and Methods

### 4.1. Sample Collection and Morphological Comparison

Based on field investigations of this putative new species, *P. xideensis*, fresh leaf samples were collected and rapidly dried in silica gel for subsequent DNA extraction and sequencing. Comprehensive photographs were taken, and voucher specimens were collected to facilitate detailed morphological comparison with its close relatives, as well as for the preparation of type material and formal species description. The voucher specimens are deposited in the Herbarium of the Chengdu Institute of Biology, Chinese Academy of Sciences (Bo Xu, xubo@cib.ac.cn), under the accession number CDBI0309739 (Figure 1). The comparison of morphological characters was undertaken using material from type localities, supplemented by digital herbarium resources (Chinese Virtual Herbarium, https://www.cvh.ac.cn/; JSTOR Global Plants, https://plants.jstor.org/) and taxonomic literature [7,8,9]. A provisional conservation status was assigned to the new species in accordance with the IUCN Red List Categories and Criteria (IUCN Standards and Petitions Committee, 2024).

### 4.2. DNA Extraction, Sequencing, Genome Assembly, and Annotation

The total genomic DNA (gDNA) was extracted from the dried leaves of *P. xideensis* using a modified CTAB method [25]. Sequencing libraries were prepared using the BGI Optimal DNA Library Prep Kit (BGI, Shenzhen, China). Sequencing was performed on a DNBSEQ-T7 platform (BGI, Shenzhen, China) to generate paired-end 150 bp reads. To obtain high-quality sequences, fastp v0.23.2 was used to filter out all low-quality reads, including those with adapters, more than 20% of bases having Phred quality < 5, and reads with >10% N content [26]. The chloroplast (cp) genome and nrITS were assembled using GetOrganelle v1.7.7.7.1 [27] and annotated using Plastid Genome Annotator (PGA) [28]. The final circular map was drawn using CPGView [29].

### 4.3. Comparative Genomics and Sequence Divergence Analysis

To compare the newly described species with other *Primula* species from a chloroplast genome perspective, we downloaded the complete chloroplast genome sequences of seven additional *Primula* sect. *Aleuritia* species from the NCBI database, resulting in a total of eight genomes (including the new species) for comparative analysis (Table 2). To investigate the contraction and expansion of the inverted repeat (IR) regions among these eight chloroplast genomes of *Primula* and its closely related species, we analyzed the IR/single-copy (IR/SC) boundaries using CPJSdraw v1.0 [30]. Furthermore, to detect potential structural variations such as gene rearrangements or inversions, synteny analysis of the *Primula* chloroplast genomes was performed using mVISTA [31,32] and Geneious v11 [33]. A preliminary multiple alignment of the eight complete *Primula* chloroplast genomes was conducted using MAFFT v7 [34]. Subsequently, nucleotide diversity (Pi) of *Primula* was quantified via a sliding window analysis (window length: 600 bp; step size: 200 bp) in DnaSP v6.12.03 [35] to identify hypervariable loci with potential phylogenetic utility. Relative synonymous codon usage (RSCU) was calculated using CPStools [36]. RSCU values greater than 1 indicate codons used more frequently than expected, values less than 1 indicate the opposite, and a value of 1.00 indicates no codon usage bias.

### 4.4. Phylogenetic Relationship Reconstruction

Phylogenetic analyses were conducted separately using whole chloroplast genomes and nuclear ribosomal internal transcribed spacer (nrITS) sequences. The chloroplast dataset consisted of *P. xideensis* assembled in this study and 62 sequences obtained from NCBI (two outgroup taxa: *Androsace limprichtii* Pax & K. Hoffm. and *Lysimachia medogensis* F. H. Chen & C. M. Hu), while the nrITS dataset included *P. xideensis*, *Primula stenocalyx* Maxim. assembled in this study, and 73 additional nrITS sequences from NCBI (outgroup taxa: *A. limprichtii*). The best-fitting nucleotide substitution models were selected with jModelTest 2.1.10 [37]: TVM+F+I+R4 for the maximum likelihood (ML) tree and GTR+F+I+G4 for the Bayesian inference (BI) tree in the chloroplast analysis, and SYM+I+G4 for both trees in the nrITS analysis. Phylogenetic trees were reconstructed using IQ-TREE 2.1.4 (ML) [38] and MrBayes 3.2.7a (BI) [39], with *Androsace limprichtii* and *Lysimachia medogensis* set as outgroups for the chloroplast trees, and *A. limprichtii* for the nrITS trees. All resulting trees were visualized and refined using the online tool tvBOT [40].

## 5. Taxonomic Treatment

*Primula xideensis* W.B.Ju, Hu Ju, Bo Xu *sp. nov.* (Figure 1, Figure 2, Figure 3 and Figure 4).

### 5.1. Type

China. Sichuan: Ejize’e Village, Mianshan Town, Xide County, Sichuan Province, growing in moist rock crevices covered with moss, 28°29′45.19″ N, 102°22′01.82″ E, 3525 m, 7 July 2023, W.B.Ju, JWB1247 (holotype CDBI!; isotypes KUN!, PE!).

### 5.2. Diagnosis

*Primula xideensis* is most similar to *P. stenocalyx*, *P. farinosa s.l.* and *P. pulchella*, but differs by its roots densely covered with multicellular hairs, leaves with dentate to irregularly deeply dentate margins, both surfaces efarinose, densely short-hairy and scabrous, corolla mouth densely white-farinose, pin flowers with stamens inserted about the middle of the corolla tube and styles adjacent to the corolla throat, thrum flowers with stamens positioned near the corolla throat and styles inserted about the middle of the corolla tube, and an oblong capsule shorter than the persistent calyx.

### 5.3. Description

**Perennial herb**, 8–18 cm tall. **Rhizome** short and stout, covered at base with numerous withered leaves from the previous year, lacking scales. **Roots** are fibrous, and adventitious roots are numerous, 3–10 cm long, densely covered with multicellular hairs. **Leaves** in an open rosette; leaf blade elliptic to obovate, 1.0–3.0 cm × 1.0–1.7 cm, apex rounded to obtuse, base attenuate, margin dentate to irregularly deeply dentate, not revolute, both surfaces efarinose, densely covered with short hairs, and scabrous; midveins and lateral veins are prominent abaxially, lateral veins 5–8 pairs; indistinct petiole, up to 0.5–1 cm long. **Scape** solitary, arising from the leaf rosette, 5–15 cm tall, glandular-farinose, apex conspicuously covered with white farina. Inflorescence an umbel, 1-whorled, with 2–6 flowers; bracts lanceolate, 6–12 mm long, apex acuminate, glandular and farinose; pedicels 8–12 mm long, glandular and densely farinose. **Calyx** tubular, 5-ribbed, 8–14 mm long, equal to or slightly shorter than the corolla tube, externally glandular, internally densely covered with white farina, base inflated in fruit, lobed to the middle, lobes oblong to lanceolate, apex acute to obtuse. **Corolla** purple-red; corolla tube 8–16 mm long; around the mouth of the corolla tube densely white-farinose; limb 1.5–2.5 cm in diameter; lobes 5, broadly obovate, emarginate at apex to 1/5 of their length. **Pin flowers** with a corolla tube 8–14 mm long; stamens inserted near the middle of the tube; style equalling or slightly exserted from the tube. **Thrum flowers** with a corolla tube 10–16 mm long; stamens attached near the corolla throat; style inserted near the middle of the tube or slightly longer. Ovary globose, glabrous; ovules numerous. **Capsule** oblong, 6–8 mm long, shorter than the persistent calyx. **Seeds** numerous, irregularly globose to pyriform, 0.5–1.0 mm in diameter.

## 6. Conclusions

In this study, we describe *Primula xideensis* as a new species from Sichuan, China, based on integrated morphological and molecular evidence. Morphological comparisons clearly distinguish it from its close allies *P. stenocalyx*, *P. farinosa s.l.*, and *P. pulchella* by a combination of root indumentum, leaf margin and surface characteristics, corolla mouth farinose condition, and relative positions of stamens and styles. Phylogenetic analyses using both complete chloroplast genomes and nrITS sequences consistently place the new species within *Primula* sect. *Aleuritia* with strong support. Comparative chloroplast genomics among eight sect. *Aleuritia* species reveal high structural conservation, yet identify several hypervariable regions (e.g., *trnK-rps16* intergenic spacer and *ycf1*) that hold potential as molecular markers for future species delimitation and population genetics. The discovery of *P. xideensis* not only adds to the rich biodiversity of the Hengduan Mountains region but also highlights the necessity of integrative taxonomy in resolving species boundaries within rapidly radiating lineages. Given its currently known restricted distribution, a conservation status of Data Deficient (DD) is proposed, urging further field surveys to determine its true range and potential threats.

## Figures and Tables

**Table 1:** Comparison of morphological characters among *Primula xideensis*, *P. stenocalyx* and *P. pulchella.*

Characters	*P. xideensis*	*P. stenocalyx*	*P. farinosa*	*P. pulchella*
Root	densely covered with multicellular hairs	Glabrous	Glabrous	Glabrous
Leaves	leaf blade elliptic to obovate	leaf blade obovate to oblanceolate or spatulate	leaf blade oblong-obovate to oblong-lanceolate	leaf blade lanceolate to linear-lanceolate or oblanceolate
	margin dentate to irregularly deeply dentate and not revolute	margin denticulate to occasionally nearly entire, usually narrowly revolute, apex obtuse or occasionally acute	margin remotely denticulate to nearly entire	margin denticulate to occasionally nearly entire, usually narrowly revolute
	both surfaces efarinose, densely covered with short hairs, and scabrous	both surfaces efarinose, only with small glands, or lower surface covered with white or yellow farina	both surfaces farinose or efarinose, glabrous	upper surface glabrous, lower surface densely covered with bright yellow or creamy yellow farina
Bracts	narrowly lanceolate, 6–12 mm long, as long as or slightly shorter than the pedicels	narrowly lanceolate, 5–10(–15) mm long, as long as to slightly longer than the pedicels	narrowly lanceolate to acuminate-subulate, 3–8 mm long, shorter than the pedicels	linear to linear-lanceolate, 3–8(–10) mm long, shorter than the pedicels
Calyx	tubular	tubular	campanulate	campanulate
	8–14 mm long, equal to or slightly shorter than the corolla tube	6–10 mm long, 2/3 as long as corolla tube	4–6 mm long, ca. equal to or slightly shorter than the corolla tube	4–8(–10) mm, 1/2 or nearly 2/3 as long as corolla tube
	parted to the middle, lobes oblong to lanceolate	divided for 1/3 or nearly 1/2 of its length, lobes oblong to lanceolate, ciliolate	parted to 1/3–1/2, lobes ovate-oblong to triangular, short ciliate	divided to or somewhat beyond the middle, lobes lanceolate to narrowly lanceolate
Corolla	corolla tube 8–16 mm long, densely white-farinose at the mouth	corolla tube 9–15 mm long, mouth not farinose	corolla tube 5–6 mm long, mouth not farinose	corolla tube 8–12 mm long, mouth not farinose
Stamens	pin flower: stamens inserted at mid-corolla tube; thrum flower: stamens positioned adjacent to corolla throat	pin flower: stamens inserted about 2 mm from base of corolla tube; thrum flower: stamens inserted slightly above middle of corolla tube.	pin flowers: stamens at middle of corolla tube; thrum flowers: stamens in upper 1/2 of corolla tube	pin flower: stamens inserted about 2 mm from base of corolla tube; thrum flower: stamen apices approaching corolla throat
styles	pin flower: styles positioned adjacent to corolla throat; thrum flower: styles inserted at mid-corolla tube	stamens and style in reciprocal positions	stamens and style in reciprocal positions	stamens and style in reciprocal positions
Capsule	oblong, shorter than the persistent calyx	oblong, nearly as long as calyx	cylindric, slightly longer than the calyx	oblong, slightly longer than to twice as long as the calyx

**Table 2:** Characteristics of 8 *Primula* complete chloroplast genomes.

Species	GenBank Accession Number	Length (bp)				GC Content (%)				Number of Genes			
		Total	LSC	SSC	IR	Total	LSC	SSC	IR	Total	CDS	tRNA	rRNA
*P. farinosa*	PP541897	154,656	85,177	17,729	25,875	37	34.8	30.3	42.7	132	87	37	8
*P. fistulosa*	PQ412516	154,368	84,776	17,824	25,884	36.9	34.8	30.2	42.7	131	86	37	8
*P. flava*	PQ536122	154,347	84,915	17,750	25,841	37	34.9	30.3	42.7	130	86	36	8
*P. gemmifera*	NC_053590	154,650	85,197	17,789	25,832	36.8	34.7	30.1	42.7	129	85	36	8
*P. pulchella*	NC_050246	154,599	83,953	17,803	25,853	36.9	34.8	30.3	42.7	130	85	37	8
*P. pumilio*	NC_065465	154,705	85,142	17,865	25,849	36.9	34.7	30.2	42.7	131	86	37	8
*P. stenocalyx*	NC_058249	153,678	84,235	17,747	25,848	36.9	34.8	30.3	42.7	132	87	37	8
*P. xideensis*	PX910298	154,324	84,969	17,661	25,847	36.9	34.8	30.2	42.7	131	86	37	8

**Table 3:** Summary of gene contents present in the *P. xideensis* chloroplast genomes.

Category	Gene Group	Gene Name
Photosynthesis related genes	Subunits of photosystem I	*psaA*, *psaB*, *psaC*, *psaI*, *psaJ*
	Subunits of photosystem II	*psbA*, *psbB*, *psbC*, *psbD*, *psbE*, *psbF*, *psbH*, *psbI*, *psbJ*, *psbK*, *psbL*, *psbM*, *psbN*, *psbT*, *psbZ*
	Subunits of NADH dehydrogenase	*ndhA* *, ndhB *(2), *ndhC*, *ndhD*, *ndhE*, *ndhF*, *ndhG*, *ndhH*, *ndhI*, *ndhJ*, *ndhK*
	Subunits of cytochrome b/f complex	*petA*, *petB* *, *petD* *, *petG*, *petL*, *petN*
	Subunits of ATP synthase	*atpA*, *atpB*, *atpE*, *atpF* *, *atpH*, *atpI*
	Large subunit of rubisco	*rbcL*
Self-replication	Proteins of large ribosomal Subunit (LSU)	*rpl14*, *rpl16* *, *rpl2* * *(2)*, *rpl20*, *rpl22*, *rpl23(2)*, *rpl32*, *rpl33*, *rpl36*
	Proteins of small ribosomal Subunit (SSU)	*rps11*, *rps12* ** *(2)*, *rps14*, *rps15*, *rps16* *, *rps18*, *rps19*, *rps2*, *rps3*, *rps4*, *rps7(2)*, *rps8*
	Subunits of RNA polymerase	*rpoA*, *rpoB*, *rpoC1* *, *rpoC2*
	Ribosomal RNAs	*rrn16(2)*, *rrn23(2)*, *rrn4.5(2)*, *rrn5(2)*
	Transfer RNAs	*trnA-UGC* * *(2)*, *trnC-GCA*, *trnD-GUC*, *trnE-UUC*, *trnF-GAA*, *trnG-UCC* * *(2)*, *trnH-GUG*, *trnI-CAU(2)*, trnI-GAU *(2), *trnK-UUU* *, *trnL-CAA(2)*, *trnL-UAA* *, *trnL-UAG*, *trnM-CAU*, *trnN-GUU(2)*, *trnP-UGG*, *trnQ-UUG*, *trnR-ACG(2)*, *trnR-UCU*, *trnS-GCU*, *trnS-GGA*, *trnS-UGA*, *trnT-GGU*, *trnT-UGU*, *trnV-GAC(2)*, *trnV-UAC* *, *trnW-CCA*, *trnY-GUA*, *trnfM-CAU*
Other genes	Maturase	*matK*
	Protease	*clpP* **
	Envelope membrane protein	*cemA*
	Acetyl-CoA carboxylase	*accD*
	c-type cytochrome synthesis gene	*ccsA*
	other	*pafI* **, *pafII*
unknown function genes	Conserved open reading frames	*ycf1*, *ycf2(2)*, *ycf15(2)*

Intron-containing genes are marked by asterisks (*), * gene with one intron; ** gene with two introns.

## Data Availability

The chloroplast genome sequence of *Primula xideensis* and the nrITS sequences of *P. xideensis* and *P. stenocalyx* have been deposited in the NCBI GenBank database under accession numbers PX910298 (chloroplast genome), PX963660 (nrITS of *P. xideensis*), and PX963661 (nrITS of *P. stenocalyx*). These data are publicly available at https://www.ncbi.nlm.nih.gov/.

## Supplementary Materials

The following supporting information can be downloaded at: https://www.mdpi.com/article/10.3390/plants15121829/s1, Table S1: Pi values of 8 Primula chloroplast genomes; Table S2: RSCU values of 8 Primula chloroplast genomes.
